# Isoniazid—Loaded Albumin Nanoparticles: Taguchi Optimization Method

**DOI:** 10.3390/polym13213808

**Published:** 2021-11-04

**Authors:** Yerkeblan Tazhbayev, Aldana Galiyeva, Tolkyn Zhumagaliyeva, Meiram Burkeyev, Bakhytgul Karimova

**Affiliations:** Department of Organic Chemistry and Polymers, Buketov Karaganda University, Karaganda 100028, Kazakhstan; zhumagalieva79@mail.ru (T.Z.); m_burkeev@mail.ru (M.B.); b.karimova2011@mail.ru (B.K.)

**Keywords:** proteins, biopolymers, bovine serum albumin, nanoparticles, isoniazid, Taguchi method, anti-TB drugs

## Abstract

Tuberculosis is one of the dangerous infectious diseases, killing over a million people worldwide each year. The search for new dosage forms for the treatment of drug-resistant tuberculosis is an actual task. Biocompatible polymer nanoparticles, in particular bovine serum albumin (BSA), are promising drug carriers. Nanoparticle (NP) parameters such as diameter, polydispersity, bioactive substance loading, and NP yield are very important when it comes to drug transport through the bloodstream. The most well-known and widely used first-line anti-tuberculosis drug, isoniazid (INH), is being used as a drug. BSA-INH NPs were obtained by an ethanol desolvation of an aqueous protein solution in the drug presence. The peculiarity of the method is that natural components, namely urea and cysteine, are used for the stabilization of BSA-INH NPs after desolvation. The characteristics of the obtained BSA-INH NPs are significantly affected by the concentration of protein, isoniazid, urea, and cysteine in the solution. The aim of the present study is to investigate the concentration effect of the system reacting components on the parameters of the NPs that are obtained. We have chosen the concentrations of four reacting components, i.e., BSA, isoniazid, urea, and cysteine, as controlling factors and applied the Taguchi method to analyze which concentration of each component has an important effect on BSA-INH NPs characteristics.

## 1. Introduction

For thousands of years, humanity has been haunted by a dangerous disease called tuberculosis. Tuberculosis is an infectious disease caused by Mycobacterium tuberculosis (MTB) [1]. This disease usually affects the lungs, but it can also affect other organs and systems. MTB is transmitted through airborne droplets by talking, coughing, and sneezing [2]. The World Health Organization (WHO) estimates that 10 million people became ill with and ~1.4 million died from tuberculosis in 2019 [3]. Nearly one out of four people on the planet is infected with Koch’s bacillus [4,5], which activates when a person’s immunity is reduced. Previously, the disease was considered to be a poverty disease and was predominantly found in underdeveloped countries. However, tuberculosis re-emerged in Western Europe with the advent of HIV advent. Tuberculosis patients are among the most vulnerable groups of the population because their lungs are affected, and their immune system is weakened. With COVID-19 disease, such patients have a high chance of developing lung failure and death [6].

Treatment for tuberculosis is a long-term process; the medicines are taken for several months or more. Because the drugs must be taken for a long time, some patients have side effects resulting from the medicine. If we classify drugs by activity, the most effective drugs are isoniazid (INH) and rifampicin (RIF). Isoniazid has been used since the beginning of the 20th century, and in some cases, MTB has mutated to become resistant to this drug, as it has to many other first-line drugs [7,8,9]. Multidrug resistance tuberculosis (MDR-TB) is much harder and longer to treat, and more than 40% of patients die. One possible way to solve the problem of targeted drug delivery, prolonging its action and possibly overcoming MDR, is the use of polymeric nanoparticles as drug delivery systems [10]. Bovine serum albumin (BSA) is one of the most promising drugs carriers in the NP form. It is a biodegradable, non-toxic carrier that can be metabolized in vivo with the formation of harmless degradation products that are bioavailable, easily purified, and soluble in water, which facilitates delivery by injection [10,11]. Albumin is already used as a drug carrier (Levemir^®^ FlexPen^®^), including in the form of nanoparticles when being used in cancer treatment (Abraxane^®^). Albumin-based nanoparticles are mostly produced using glutaraldehyde for macro- chain cross-linking. We were the first to propose a method of obtaining BSA nanoparticles using natural components such as urea and L-cysteine [12,13]. In this case, urea plays the role of a chaotropic agent, which unfolds albumin chains and increases the availability for interaction with cysteine. As a result, the thiol-disulfide exchange reaction between BSA NPs macro- chains is stabilized [12,13,14]. In addition, the authors of the present work [15] have shown that cysteine can play a positive role in overcoming MDR. The INH/cysteine combination increases INH activity in macrophages and increases MTB oxygen consumption, which may contribute to the bactericidal activity of the INH/cysteine combination [15].

Particle sizes are of great importance in drug transport applications. This is due to the NP size limitation for injectable drug forms. In addition, several researchers have shown that they can be localized in different parts of the human respiratory system depending on the particle diameter [16]. At the same time, the parameters and yield of nanoparticles and the drug loading efficiency depend on many factors, namely the concentration of urea, cysteine, isoniazid, and bovine albumin. The aim of this work is to develop and optimize the conditions for obtaining BSA nanoparticles and for immobilizing the INH in them. A controlled INH delivery system with high drug loading and encapsulation efficiency as well as NP yield should be obtained. The effect of different concentrations of albumin, isoniazid, urea, and cysteine was investigated in order to optimize the nanoparticle production method. The parameters were optimized using Taguchi method by considering equal contributions of the variables [17,18,19]. The optimization of such significant parameters such as particle size estimation, encapsulation efficiency, and drug loading into the polymer matrix as well as NP yield was performed to solve that problem.

## 2. Materials and Methods

### 2.1. Materials

Bovine serum albumin (lyophilized powder, 98%) (BSA), isoniazid (INH) with indicated purity over 99%, and L-cysteine (98.5%) were purchased from Sigma Aldrich (Taufkirchen, Germany). Absolute ethanol was purchased from DosFarm (Almaty, Kazakhstan). Urea (99.5%) was purchased from HimPribor-SPb (Saint Petersburg, Russia).

### 2.2. Preparation of BSA NPs

BSA-derived nanoparticles (BSA NPs) were prepared by a desolvation method in accordance with a slightly modified procedure described elsewhere [12,13]. Briefly, the pre-specified amount of serum albumin powder (0.03, 0.06 or 0.12 g) was dissolved in 3 mL of distilled water (pH = 7.4) by stirring at 200 rpm, avoiding clumping and foaming, at 23 °C for 10 min. The concentration of these prepared protein solutions was 10, 20, and 40 mg/mL, respectively. Then, 0.5 mL of aqueous urea solution (its concentration was 6, 7 or 8 M) was added and sonicated using an ultrasonic bathtub (Launch Tech, Shenzhen, China) for 3 min. After that, 16 mL of ethanol was added to each protein solution at a rate of 1 mL/min, thus resulting in the turbid dispersion of albumin nanoparticles. Then, 3 mL of an aqueous solution of l-cysteine, in which the concentration of the amino acid was 1, 2.5, or 5 mg/mL, was introduced. The resultant nanoparticle suspension was rinsed using three centrifugation steps (MiniSpi, Eppendorf, Hamburg, Germany) at 14,000 rpm for 15 min each to remove dissolved substances and ethanol from the mixture. The precipitated nanoparticles were re-dispersed in 10 mL of deionized water after each phase of centrifugation using an ultrasonic bathtub; the ultrasound period was 10 min.

### 2.3. Preparation of BSA-INH-NPs

A pre-prepared isoniazid solution was added to the BSA-NPs that were obtained so that the concentrations of the drug in the system were 2, 4, and 6 mg/mL. The adsorption reaction was kept under constant magnetic stirring (stirring velocity was 300 rpm) for 2 h. Next, the NPs suspension was purified by three cycles of centrifugation at 14,000 rpm (Eppendorf) for 15 min to remove any isoniazid that had not been absorbed.

### 2.4. Nanoparticles Size Measurement, Zeta Potential Analysis, and Surface Morphology

The average particle size of the BSA-NPs, both with and without isoniazid, was measured by dynamic light scattering (DLS) using a particle size analyzer (Zetasizer NanoZS90, Malvern Instruments Limited, Worcestershire, UK). The samples were diluted with deionized water and were measured at a scattering angle of 90° and a temperature of 25 °C. The polydispersity index (PDI) gave an estimate of the BSA-NPs size distribution. The size, shape, and surface morphology of the BSA-NPs were examined by scanning electron microscopy (MIRA 3LM TESCAN, Brno, Czech Republic, and EU).

### 2.5. Encapsulation Efficiency and Production Yield

After centrifugation while the nanoparticles were being washed, the supernatant was collected, and the drug amount in the solution was determined using the high-performance liquid chromatography (HPLC) method (Shimadzu LC-20 Prominence). The amount of drug encapsulated in the nanoparticles was then determined by subtracting the amount in the supernatant (free drug) that was not entrapped in the nanoparticles. Encapsulation efficiency and production yield were calculated as follows:

$$\mathrm{Encapsulation}\;\mathrm{efficiency}\;\left(\mathrm{EE}\%\right)=\frac{\mathrm{Mass}\;\mathrm{of}\;\mathrm{the}\;\mathrm{total}\;\mathrm{Drug}\;-\mathrm{Mass}\;\mathrm{of}\;\mathrm{free}\;\mathrm{Drug}}{\mathrm{Mass}\;\mathrm{of}\;\mathrm{total}\;\mathrm{Drug}}\times 100\%$$

$$\mathrm{Production}\;\mathrm{Yield}\;\left(\%\right)=\frac{\;\mathrm{Mass}\;\mathrm{of}\;\mathrm{total}\;\mathrm{nanoparticles}}{\mathrm{Mass}\;\mathrm{of}\;\mathrm{the}\;\mathrm{total}\;\mathrm{Drug}+\mathrm{Mass}\;\mathrm{of}\;\mathrm{total}\;\mathrm{BSA}}\times 100\%$$

### 2.6. Determination of the In Vitro INH Drug Release

Drug release experiments were performed “in vitro” to evaluate the release behavior of INH from the NPs. NPs (24 mg) were re-dispersed in 14 mL of phosphate-buffered saline (PBS, pH 7.4) and were incubated at 37 °C under stirring at 200 rpm. At different times, 1 mL of the release medium was removed. The aliquots that were removed were centrifuged, and supernatant containing the drug INH was analyzed by HPLC (excitation 262 nm); all of the measurements were performed in triplicate. The release was measured at intervals of 0, 2, 4, 8, 12, and 24 h. The cumulative drug release was plotted against time.

### 2.7. Taguchi Design of Experiment (DoE)

The Taguchi DoE method, which is suitable for studying a large number of factors, was used to evaluate the influence of the optimization parameters and to minimize the number of experiments using the statistical software Design Expert (version 13, Stat-Ease, Minneapolis, MN, USA). Four factors, namely BSA, isoniazid, urea, and cysteine concentrations were evaluated by constructing and using an orthogonal array (OA) L9 (Table 1). This scheme was applied to identify the significant factors that affect the size, PDI, and production efficiency of obtaining BSA-INH NPs. Three different levels and a fractional factor design were derived for each parameter, particularly the standard L9 OA (Table 1).

## 3. Results and Discussions

### 3.1. Optimization of Nanoparticles Preparation

**Figure 1:**
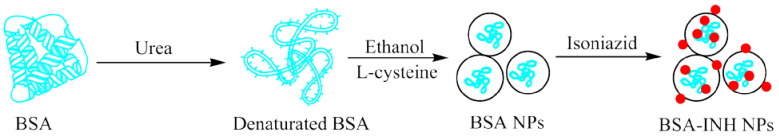
Scheme for obtaining BSA-INH NPs.

The desolvation procedure is a commonly used method for producing protein-based nanoparticles (NPs) [20,21,22]. The desolvation of bovine serum albumin (BSA) with ethanol leads to the production of well-defined nanoparticles with denatured albumin and the formation of a matrix of spheres [23]. Glutaraldehyde was mainly used as a cross-linking agent to prepare albumin-based nanoparticles in previous studies [24,25]. Tazhbayev et al. presented a method that does not need the use of a synthetic stabilizer, replacing it with natural agents such as urea and cysteine [12,13]. However, the process parameters have a great influence on size, size distribution, and the obtained BSA nanoparticles yield. BSANPs containing isoniazid (INH) were prepared by the desolvation method, in which urea was used as a denaturing agent and where ethanol was used as a desolvating agent, followed by the L-cysteine reduction step. INH immobilization was performed by drug adsorption onto the surface of the albumin nanoparticles that were obtained (Figure 1).

It is fair to say that a denatured protein is not completely identical in its physico-chemical and biological properties to a native protein [26]. There may be an increase in the reactivity of certain chemical groups in the protein that were unable to be detected before denaturation. This may be a reflection of the nature of the drug’s interaction with protein molecules. The study of the biological properties of denatured proteins is a task for a separate study and was not considered within the framework of this work. The task here was to find a synthesis methodology for an effective and safe drug carrier. The presence of drugs based on denatured serum albumin (Abraxane^®^, Levemir^®^, FlexPen^®^) in medical practice gives hope that there are no limiting factors when using BSA to immobilise INH.

A Taguchi orthogonal arrays (OA) design was used to determine the optimal conditions and to select the parameters with the greatest influence on the BSA nanoparticle size. Nine experiments were performed to evaluate the optimal conditions for nanoparticle synthesis. The structure of the OA design and the measurements results of particle size and PDI are shown in Table 2. The particle size for each nanoparticle sample was determined by DLS. The encapsulation efficiency (EE) and the nanoparticles yield were also calculated. 

Particle size, PDI, and production efficiency of BSA-INH NPs were analyzed in Design Expert software. An ANOVA table was used to determine the process parameters that significantly affected the properties of BSA-INH NPs. The ANOVA table for the average particle size is presented in Table 3. Thus, the raw data were analyzed by ANOVA, and it was determined that the l-cysteine concentration had no effect on the average particle size and PDI. Therefore, this factor was not considered in the ANOVA analysis. For the particle size, *p*-values less than 0.500 indicate the significance of the model conditions. Albumin and urea concentrations are significant model conditions, whereas isoniazid concentration can be considered as a condition that is not significant. If we remove the non-significant variable, we can improve the model. Even though INH concentration has a *p*-value of 0.0840, it was still included in the model because the model becomes significant when this parameter is kept. The R^2^ value calculated with help when the ANOVA is 0.989, which means that about 99% of the variability in the data can be explained by the model [17]. The predicted R^2^ meaning equal to 0.77 is in reasonable agreement with the adjusted R^2^ value (0.955); i.e., the difference is less than 0.2. Adeq Precision measures the signal-to-noise ratio. A ratio that is greater than 4 is a desirable. Our ratio is equal to 15.83, which points to an adequate signal. This model can be used to navigate the design space.

To identify the PDI, the values that were analyzed were similar to the average particle size. In the ANOVA table (Table 4), the *p*-values for the model are greater than 0.05, which indicates that the model is not significant. The F-value of the model is 1.36, which indicates that the model is not significant with respect to noise. The probability that such a large F-value could be due to noise is 48.23%. In this case, the concentrations of albumin, urea, and isoniazid are all significant conditions that can be used to support the model structure because we did not consider the effect of noise (temperature, rate of ethanol addition, etc.) on the PDI values of the nanoparticles obtained in the nanoparticle production process.

**Figure 2:**
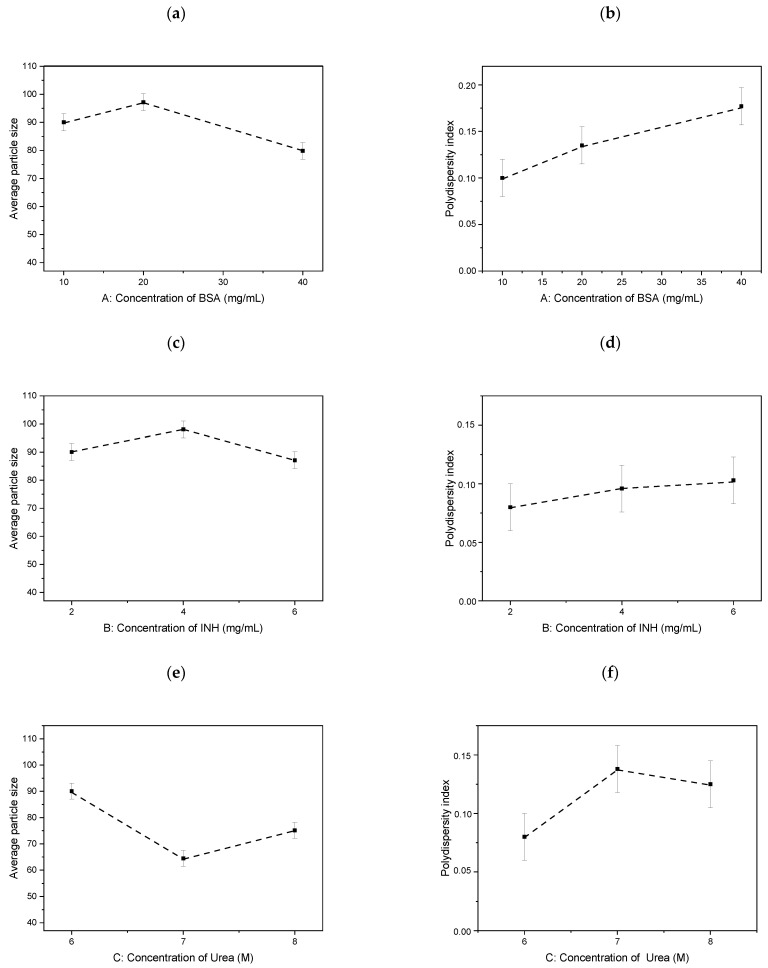
Influence of parameters on particle size: (**a**) Concentration of BSA, (**c**) Concentration of INH, (**e**) Concentration of Urea and polydispersity: (**b**) Concentration of BSA, (**d**) Concentration of INH, (**f**) Concentration of Urea.

Figure 2 shows the different influence of factors on nanoparticle size and PDI. The NP size decreases with increasing concentrations of albumin and isoniazid as well as when urea with a concentration equal to 7 M is applied. The PDI increases with increasing concentrations of basic substances.

Similarly, the EE and yield of BSA-INH NPs were analyzed as input data in Design Expert software, and the analysis results are presented in Table 5 and Table 6. Production efficiency is mostly influenced by the concentration of BSA and isoniazid, so these parameters appear in the ANOVA analysis. When analyzing the parameters of the degree of albumin binding to the anti-TB drug, the meaning of the model’s F-value is 6.99, and the *p*-value is 0.043, so the model is significant. The calculated meaning of R^2^ is equal to 0.875, which is not as close to the corrected R^2^ equal of 0.75 as one would expect; that is, the difference is no more than 0.2. Therefore, we used this model for further investigation. The Adeq Precision ratio is 7.32, which indicates an adequate signal. This model can be used to navigate the design space.

In analyzing the parameters for the BSA-INH NP yield (Table 6), the model has an F-value of 3.3, and the *p*-value for the model is 0.137, so the model is not significant with respect to noise. The probability that such a large F-value could occur due to noise is 13.71%. The predicted R^2^ value of 0.969 is not as close to the adjusted R^2^ value of 0.876 as one would expect. This could indicate a large block effect or a possible problem with the model and/or data. However, the difference in the two values is not too large (about 0.2), so we still used this model for further analysis. The adjusted R^2^-value is especially useful for comparing models with different numbers of terms. The Adeq Precision ratio is equal to 15.83 and indicates an adequate signal. This model can be used to navigate the design space.

**Figure 3:**
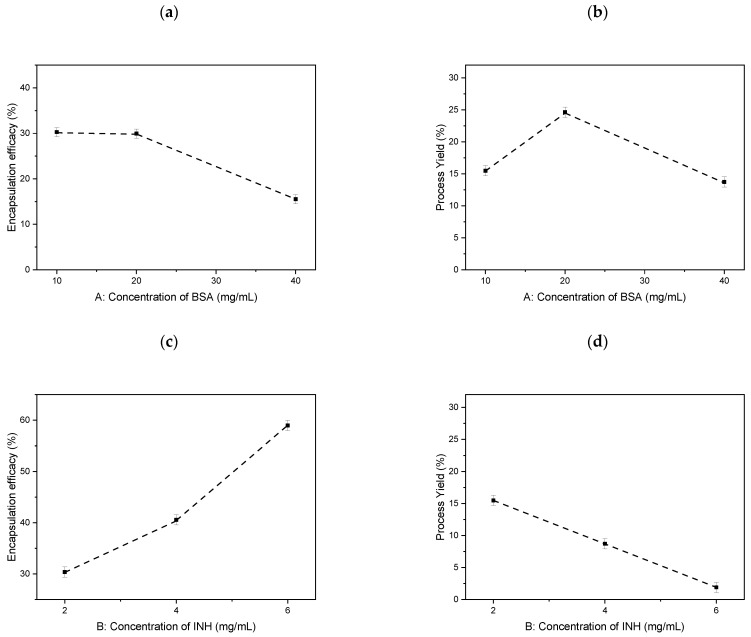
Influence of parameters on EE: (**a**) Concentration of BSA, (**c**) Concentration of INH and NPs yield: (**b**) Concentration of BSA, (**d**) Concentration of INH.

Figure 3 shows the different effects of the independent variables on the EE and NPs yield. The EE decreases with increasing albumin concentration and increases with increasing isoniazid concentration. The yield of nanoparticles increases with increasing albumin concentration and decreases with increasing isoniazid concentration.

Process parameters were selected for computer optimization using the Design Expert software. Table 7 shows the specific optimum conditions for BSA-INH NPs. In fact, our aim is to obtain nanoparticles with a minimum size and PDI and with maximum production efficiency at optimal conditions.

Based on the results obtained by ANOVA, the Design Expert software proposed optimal solutions for obtaining BSA nanoparticles immobilized with INH (Table 8). Optimization was performed using a desirability function to obtain the levels of the investigated factors that minimized the particle size and PDI while also maximizing the production efficiency [17]. The predicted optimal factors of Solution 1 with a desirability factor equal to 42.5% were chosen to prepare INH-loaded BSA nanoparticles, although they do not provide the lowest PDI and larger size, but they have a high EE and NP yield. The nanoparticles were then prepared using Solution 1 from Table 8.

Table 9 shows a comparison of the predicted particle size and PDI as well as the production efficiency with the experimental results using the optimal conditions. The differences between the predicted and experimental data were observed. The experimentally obtained PDI data are lower than those obtained in the model. This means that the nanoparticles are rather monodisperse ones. The binding degree values as well as the nanoparticle yield are higher than the predicted ones. We assume that the high values may be the result of the transformation of the results required for prediction. This study shows that the size and production efficiency of synthesized BSA nanoparticles can be improved using the Taguchi method.

### 3.2. Physical-Chemical Characteristics of Bovine Albumin Nanoparticles Immobilized with Isoniazid

**Figure 4:**
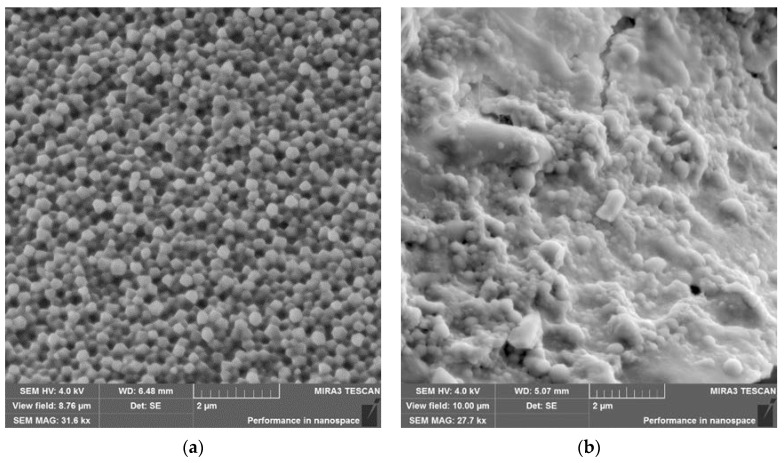
Microscopic images: (**a**) SEM image of BSA-NPs, (scale = 2 µm) and (**b**) SEM image of BSA-INH NPs (scale = 2 µm).

Morphological analysis of the BSA NPs and BSA-INH NPs samples was performed using a scanning electron microscope (SEM), and the obtained images are shown in Figure 4. Both types of synthesized NPs have a spherical morphology and an average size of less than 200 nm (108.6 ± 3 nm and 98.8 ± 1 nm, respectively). When the drug substance is immobilized, the particle shape is slightly changed, deviating more and more from the regular shape but retaining also roundness.

**Figure 5:**
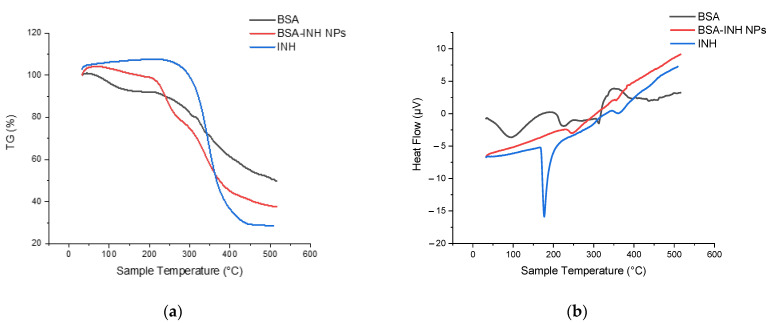
Thermogravimetric (**a**) and differential scanning calorimetry (**b**): INH; BSA; BSA-INH NPs.

A thermogravimetric analysis (TGA) (Figure 5a) and differential scanning calorimetry (DSC) (Figure 5b) of the individual components of the system and the obtained NPs were performed to confirm the inclusion of isoniazid in the BSA NPs. A characteristic endothermic peak for INH is observed at 177.2 °C, with a change in the enthalpy (Figure 5b) that is not accompanied by a mass loss and that characterizes the INH melting point. Further, an exothermic peak is observed at 345.6 °C. There is a mass loss of up to 72% (Figure 5a) in the interval of 250–430 °C, which may correspond to the drug decomposition temperature. The endothermic values for the BSA peaks at 98.9 °C, 227.3 °C, and 313 °C were recorded (Figure 5b), and since the albumin molecule consists of three different domains [27], three transitions were observed during thermal denaturation in the TGA curve. The DSC curve for the BSA-INH NPs (Figure 5b) shows an endothermic peak at 250 °C, which probably corresponds to the melting period. The TGA curve for BSA-INH NPs (Figure 5a) represents the decomposition of the protein nanoparticles within the interval 220–380 °C, with a mass loss of more than 50%. However, in comparison with the pure BSA curves, a significant movement into higher temperature regions was observed. This may be due to the influence of the drug substance.

**Figure 6:**
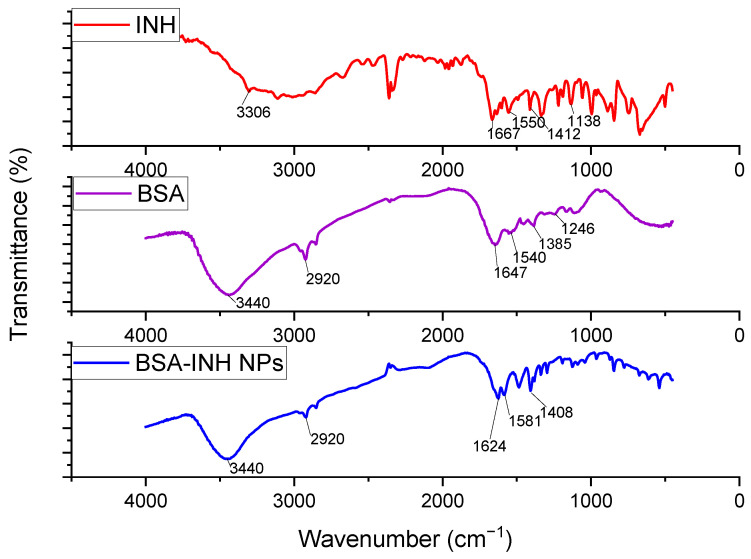
FT-IR spectra of the anti-TB drug isoniazid, BSA, and BSA NPs immobilized INH.

FT-IR spectra of isoniazid, BSA, and immobilized INH albumin nanoparticles were obtained (Figure 6). The presented spectra consist of the bands of the original albumin detected at 3440 cm^−1^ (A-amide bound to N–H), and for the next pointed peak at 2920 cm^−1^ (B-amide bound to the free ion), amide II, the recorded peak at 1540 cm^−1^ is due to C–N stretching and N–H bending vibrations. The peak of amide I at 1647 cm^−1^ corresponds to the C–O bond; the detected CH_2_ groups are located at 1385 cm^−1^, and amide III at ~1246 cm^−1^ is associated with –C–N– group stretching and N–H bending vibrations [13]. Isoniazid (Figure 6) shows characteristic absorption bands at 3306 cm^−1^ for the N-H bond, 1667 cm^−1^ (C=O) is conjugated to pyridine, 1550 cm^−1^ is assigned to ring C $\dddot{-}$ N symmetric stretching vibrations, and 1412 cm^−1^ is assigned to ring C $\dddot{-}$ C symmetric vibrations [28]. The BSA-INH-NPs spectrum shows characteristic peaks for the protein and isoniazid structure, which testifies that there was no chemical interaction between the BSA-NPs and isoniazid.

**Figure 7:**
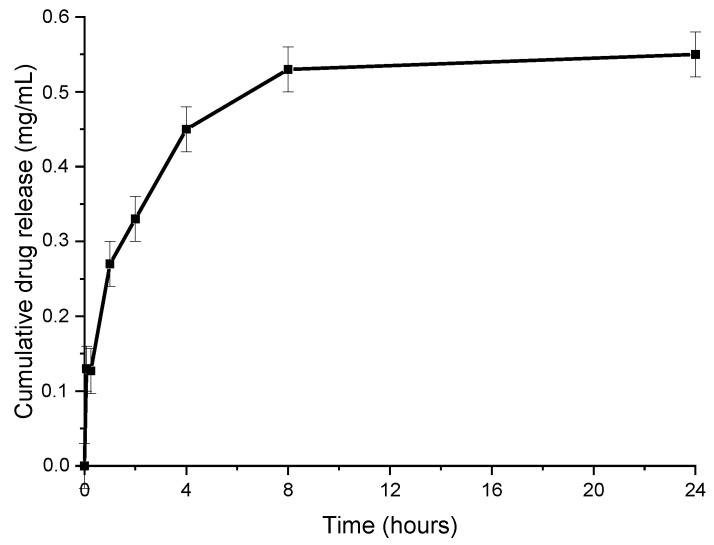
Release of isoniazid from albumin nanoparticles in phosphate-buffered saline.

The release profile of INH from BSA-INH NPs was analyzed in PBS (pH = 7.4) (Figure 7). We used the sample taken after optimization with an isoniazid concentration of 4 mg/mL. The drug was released from BSA-INH-NPs within 24 h. The initial burst release after 2 h could be due to the desorption of the adsorbed INH from the surface of the particles, which is in agreement with the results observed in [29]. BSA-INH NPs have shown a sustained release for at least 72 h when compared to the free drug, which we studied earlier in [30], and the results here demonstrated almost 100% release within 5 h.

## 4. Conclusions

Thus, a new possibility for obtaining BSA nanoparticles and their immobilization by the anti-tuberculosis drug isoniazid has been shown. Albumin nanoparticles obtained by ethanol desolvation can be stabilized in the presence of cysteine due to thiol–disulfide exchange reactions. The properties of nanoparticles are significantly affected by the concentration of BSA, urea, l-cysteine, and the drug. The application of the Taguchi method significantly reduces the time and the number of experiments needed to find the optimal conditions for obtaining BSA-INH NPs. The calculated values correlate well with the experimental characteristics of the nanoparticles. The SEM proved the circular shape of the obtained nanoparticles and the absence of coagulation during sample storage. The obtained BSA-INH NPs have satisfactory physicochemical parameters for further application as drug transport systems. With a sufficiently high degree of drug binding to the polymer (more than 50%), they remain relatively chemically inert with each other, which was proven by the TGA-DSC analysis and FT-IR results. Consequently, the BSA-INH NPs complex is quite viable and has prospects for further studies of biological activity. At present, the samples are being tested for antituberculosis activity against a number of tuberculosis strains, and the results will be published in subsequent papers.

## Figures and Tables

**Table 1:** Taguchi L9 experimental design for the production of BSA-INH NPs.

Process Parameters	Units	Level 1	Level 2	Level 3
**A: Concentration of BSA**	mg/mL	10	20	40
**B: Concentration of INH**	mg/mL	2	4	6
**C: Concentration of Urea**	M	6	7	8
**D: Concentration of L-cysteine**	mg/mL	1	2.5	5

**Table 2:** Particle size and polydispersity index (PDI) of the BSA-INH NPs obtained.

Run	A: Concentration of BSA	B: Concentration of INH	C: Concentration of Urea	D: Concentration of L-Cysteine	Size	PDI	Encapsulation Efficiency	Yield of NPs
	mg/mL	mg/mL	M	mg/mL	nm		%	%
**1**	40	2	8	2.5	66.2 ± 4	0.207 ± 0.01	16.5 ± 1.5	10 ± 1
**2**	40	4	6	5	91.6 ± 3	0.195 ± 0.02	16 ± 2	16 ± 2
**3**	20	4	8	1	90.8 ± 0.5	0.128 ± 0.02	42 ± 8	19 ± 2
**4**	10	2	6	1	90.1 ± 3	0.080 ± 0.03	28 ± 8	17 ± 1.4
**5**	10	4	7	2.5	74.9 ± 6	0.125 ± 0.03	49 ± 6	99 ± 2
**6**	20	6	6	2.5	95.9 ± 0.5	0.131 ± 0.04	56 ± 4	18 ± 1.5
**7**	10	6	8	5	75.7 ± 4	0.147 ± 0.02	53 ± 5	12 ± 3
**8**	40	6	7	1	49.9 ± 2	0.199 ± 0.01	53 ± 6	3 ± 0.3
**9**	20	2	7	5	75.8 ± 4	0.214 ± 0.01	32 ± 7	29 ± 3

**Table 3:** ANOVA table for the average particle size.

Source	Sum of Squares	Degrees of Freedom	Mean Square	F-Value	*p*-Value	
**Model**	23,221.8	6	3870.3	29.02	0.034	significant
**A: Concentration of BSA**	6680.6	2	3340.3	25.04	0.038	
**B: Concentration of INH**	2908.6	2	1454.3	10.90	0.084	
**C: Concentration of Urea**	13,632.6	2	6816.3	51.10	0.019	
**Residual**	266.8	2	133.4			
**Cor Total**	23,488.6	8				

**Table 4:** ANOVA table for the polydispersity.

Source	Sum of Squares	Degrees of Freedom	Mean Square	F-Value	*p*-Value	
**Model**	0.0022	6	0.0004	1.36	0.482	not significant
**A: Concentration of BSA**	0.0016	2	0.0008	2.99	0.25	
**B: Concentration of INH**	0.0002	2	0.0001	0.289	0.776	
**C: Concentration of Urea**	0.0004	2	0.0002	0.793	0.558	
**Residual**	0.0005	2	0.0003			
**Cor Total**	0.0027	8				

**Table 5:** ANOVA table for the encapsulation efficiency.

Source	Sum of Squares	Degrees of Freedom	Mean Square	F-Value	*p*-Value	
**Model**	2.055 × 10^6^	4	5.138 × 10^5^	6.99	0.043	significant
**A: Concentration of BSA**	3.696 × 10^5^	2	1.848 × 10^5^	2.51	0.196	
**B: Concentration of INH**	1.686 × 10^6^	2	8.428 × 10^5^	11.46	0.022	
**C: Concentration of Urea**	2.942 × 10^5^	4	73,539.39			
**Residual**	2.349 × 10^6^	8				
**Cor Total**	2.055 × 10^6^	4	5.138 × 10^5^	6.99	0.043	

**Table 6:** ANOVA table for the NPs yield.

Source	Sum of Squares	Degrees of Freedom	Mean Square	F-Value	*p*-Value	
**Model**	1.141 × 10^5^	4	28,533.96	3.30	0.137	not significant
**A: Concentration of BSA**	85,225.87	2	42,612.94	4.93	0.083	
**B: Concentration of INH**	28,909.95	2	14,454.98	1.67	0.297	
**Residual**	34,593.67	4	8648.42			
**Cor Total**	1.487 × 10^5^	8				

**Table 7:** Constraints of independent variables and responses.

Name	Goal	Lower Limit	Upper Limit
**A: Concentration of BSA**	is in range	10	40
**B: Concentration of INH**	is in range	2	6
**C: Concentration of Urea**	is equal to 7	6	8
**D: Concentration of L-Cysteine**	is equal to 2.5	1	5
**Size**	minimize	49.9	95.9
**PDI**	minimize	0.08	0.214
**EE**	maximize	16	56
**Yield**	maximize	3	29

**Table 8:** Optimum solutions for BSA-INH NP synthesis.

Name	1	2	3	4
**A: Concentration of BSA**	**20**	10	20	10
**B: Concentration of INH**	**4**	2	2	4
**C: Concentration of Urea**	**7**	7	7	7
**D: Concentration of l-Cysteine**	**2.5**	2.5	2.5	2.5
**Size**	**82.3**	66.3	74	75
**PDI**	**0.167**	0.154	0.191	0.125
**EE**	**46**	36	36	46
**Yield**	**19.6**	14	24	5
**Desirability**	**42.5**	39.8	36.9	34.2

**Table 9:** Predicted and experimental results for BSA-INH-NPs.

	Size (nm)	PDI	Encapsulation Efficiency (%)	Yield of NPs (%)
**Predicted**	82.3	0.167	46	19.6
**Experimental**	98.8 ± 1	0.068 ± 0.01	50 ± 3	26 ± 3
**Error %**	20	59.3	8.7	32.6

## Data Availability

Y.T., A.G., T.Z., M.B., B.K. 2021. https://doi.org/10.7910/DVN/HZHOVB.

## References

[1] Hershkovitz I., Donoghue H.D., Minnikin D.E., May H., Lee O.Y.-C., Feldman M., Galili E., Spigelman M., Rothschild B.M., Bar-Gal G.K. (2015). Tuberculosis origin: The Neolithic scenario. Tuberculosis.

[2] Mazlan M., Tazizi M.M., Ahmad R., Noh M., Bakhtiar A., Wahab H., Gazzali A.M. (2021). Antituberculosis Targeted Drug Delivery as a Potential Future Treatment Approach. Antibiotics.

[3] World Health Organization (2020). Global Tuberculosis Report.

[4] Hunter R.L. (2020). The Pathogenesis of Tuberculosis—The Koch Phenomenon Reinstated. Pathogens.

[5] Chapagain A., Yaqoob M.M. (2019). Tuberculosis in the 21st century: A narrative review. Néphrol. Thér..

[6] Stjepanovic M., Belic S., Buha I., Maric N., Baralic M., Mihailovic-Vucinic V. (2021). Unrecognized tuberculosis in a patient with COVID-19. Srp. Arh. Celok. Lek..

[7] Scior T., Morales I.M., Eisele S.J.G., Domeyer D., Laufer S. (2002). Antitubercular Isoniazid and Drug Resistance of Mycobacterium tuberculosis—A Review. Arch. Pharm..

[8] Narmandakh E., Tumenbayar O., Borolzoi T., Erkhembayar B., Boldoo T., Dambaa N., Burneebaatar B., Nymadawa N., Mitarai S., Jav J.S. (2020). Genetic Mutations Associated with Isoniazid Resistance in Mycobacterium tuberculosis in Mongolia. Antimicrob. Agents Chemother..

[9] Arun K.B., Madhavan A., Abraham B., Balaji M., Sivakumar K.C., Nisha P., Kumar R.A. (2020). Acetylation of Isoniazid Is a Novel Mechanism of Isoniazid Resistance in Mycobacterium tuberculosis. Antimicrob. Agents Chemother..

[10] Rather M.A., Amin S., Maqbool M., Bhat Z.S., Gupta P.N., Ahmad Z. (2016). Preparation and In Vitro Characterization of Albumin Nanoparticles Encapsulating an Anti-Tuberculosis Drug-Levofloxacin. Adv. Sci. Eng. Med..

[11] Jahanshahi M., Babaei Z. (2008). Protein nanoparticle: A unique system as drug delivery vehicles. Afr. J. Bio-Technol..

[12] Tazhbayev Y., Mukashev O., Burkeev M., Kreuter J. (2019). Hydroxyurea-Loaded Albumin Nanoparticles: Preparation, Characterization, and In Vitro Studies. Pharmaceutics.

[13] Tazhbayev Y., Mukashev O., Burkeyev M., Lozinsky V.I. (2020). Synthesis and Comparative Study of Nanoparticles Derived from Bovine and Human Serum Albumins. Polymers.

[14] Sidorskii E.V., Krasnov M.S., Yamskova V.P., Lozinsky V.I. (2020). Cryostructuring of Polymeric Systems: 57 Spongy Wide-Porous Cryogels Based on the Proteins of Blood Serum: Preparation, Properties and Application as the Carriers of Peptide Bioregulators. Gels.

[15] Vilchèze C., Hartman T., Weinrick B., Jain P., Weisbrod T.R., Leung L.W., Freundlich J.S., Jacobs W.R. (2017). Enhanced respiration prevents drug tolerance and drug resistance in Mycobacterium tuberculosis. Proc. Natl. Acad. Sci. USA.

[16] Costa A.M.M.M., Pinheiro M., Magalhães J., Ribeiro R., Seabra V., Reis S., Sarmento B. (2016). The formulation of nanomedicines for treating tuberculosis. Adv. Drug Deliv. Rev..

[17] Pham D.-D., Fattal E., Tsapis N. (2015). Pyrazinamide-loaded poly(lactide-co-glycolide) nanoparticles: Optimization by experimental design. J. Drug Deliv. Sci. Technol..

[18] Tekade R.K., Chougule M.B. (2013). Formulation Development and Evaluation of Hybrid Nanocarrier for Cancer Therapy: Taguchi Orthogonal Array Based Design. BioMed Res. Int..

[19] Kunda N.K., Alfagih I.M., Dennison S.R., Tawfeek H.M., Somavarapu S., Hutcheon G.A., Saleem I.Y. (2014). Bovine Serum Albumin Adsorbed PGA-co-PDL Nanocarriers for Vaccine Delivery via Dry Powder Inhalation. Pharm. Res..

[20] Weber C., Coester C., Kreuter J., Langer K. (2000). Desolvation process and surface characterisation of protein nanoparticles. Int. J. Pharm..

[21] Elzoghby A.O., Samy W.M., Elgindy N.A. (2012). Albumin-based nanoparticles as potential controlled release drug delivery systems. J. Control. Release.

[22] Tazhbayev Y.M., Burkeyev M.Z., Zhaparova L.Z., Zhumagaliyeva T.S., Agdarbek A.A. (2019). Albumin nanoparticles loaded with the antitumor drug «Hydroxycarbamide» by the incorporation method. Bull. Karaganda Univ. Chem. Ser..

[23] Von Storp B., Engel A., Boeker A., Ploeger M., Langer K. (2011). Albumin nanoparticles with predictable size by desolvation procedure. J. Microencapsul..

[24] Langer K., Balthasar S., Vogel V., Dinauer N., von Briesen H., Schubert D. (2003). Optimization of the preparation process for human serum albumin (HSA) nanoparticles. Int. J. Pharm..

[25] Tazhbayev Y.M., Burkeyev M.Z., Zhaparova L.Z., Zhumagalieva T.S., Arystanova Z.T., Mukhanova D.A. (2018). Preparation and characterization of empty nanoparticles of poly-D.L-lactic acid and serum albumin. Bull. Karaganda Univ. Chem. Ser..

[26] Muzammil S., Kumar Y., Tayyab S. (2000). Anion-induced stabilization of human serum albumin prevents the formation of intermediate during urea denaturation. Proteins Struct. Funct. Bioinform..

[27] Michnik A., Michalik K., Kluczewska-Gałka A., Drzazga Z. (2006). Comparative DSC study of human and bovine serum albumin. J. Therm. Anal. Calorim..

[28] Gunasekaran S., Sailatha E., Seshadri S., Kumaresan S. (2009). FTIR, FT Raman spectra and molecular structural confir-mation of isoniazid. Indian J. Pure Appl. Phys..

[29] Burkeev M.Z., Zhaparova L.Z., Tazhbaev E.M., Zhumagalieva T.S., Ali S.I., van Herk A.M. (2013). In Vitro Studies of Capreomycin Sulfate Release from Polyethylcyanoacrylate Nanoparticles. Pharm. Chem. J..

[30] Tazhbayev Y., Galiyeva A., Zhumagaliyeva T., Burkeyev M., Kazhmuratova A., Zhakupbekova E., Zhaparova L., Bakibayev A. (2021). Synthesis and characterization of isoniazid immobilized polylactide-co-glycolide nanoparticles. Bull. Karaganda Univ. Chem. Ser..

